# A case report of combined oxidative phosphorylation deficiency 35 (COXPD35) in Palestine caused by novel compound heterozygous TRIT1 variants

**DOI:** 10.1097/MD.0000000000047885

**Published:** 2026-02-28

**Authors:** Ali A.A. Doudin, Weam N.I. Omran, Saja M. Alkomi, Ahmad Rjoub, Hamza Shoubaki, Shurooq Tayseer Abu Mufreh

**Affiliations:** a Department of Pediatrics, Dura Governmental Hospital, Hebron, Palestine; b Department of Medicine, Faculty of Medicine, Al-Quds University, Jerusalem, Palestine; c Department of Medicine, College of Medicine and Health Sciences, Palestine Polytechnic University, Hebron, Palestine; d Department of Medicine, Faculty of Medicine and Health Sciences, An-Najah National University, Nablus, Palestine; e Department of Pediatrics, College of Medicine and Health Sciences, Palestine Polytechnic University, Hebron, Palestine; f Department of Radiology, Faculty of Health Professions, Al-Quds University, Jerusalem, Palestine.

**Keywords:** case report, combined oxidative phosphorylation deficiency 35 (COXPD35), mitochondrial disorder, *TRIT1*, whole-exome sequencing (WES)

## Abstract

**Rationale:**

Combined oxidative phosphorylation deficiency 35 (COXPD35) is an extremely rare mitochondrial disorder inherited in an autosomal recessive pattern. It results from pathogenic variants in the *TRIT1* gene, leading to hypomodified cytosolic and mitochondrial tRNAs. We report the first identified case of COXPD35 in Palestine, resulting from 2 novel variants of the *TRIT1* gene.

**Patient concerns:**

A 2-year-and-6-month-old female patient with seizures, neurodevelopmental delay, microcephaly, dysmorphic facial features, abnormal electroencephalogram (EEG), and thinning of the corpus callosum on Brain magnetic resonance imaging, presenting to the Emergency Department with status epilepticus, and right lower lobe pneumonia.

**Diagnoses:**

Combined oxidative phosphorylation deficiency 35 (COXPD35) caused by 2 novel *TRIT1* variants that have never been previously reported in the literature, diagnosed clinically, and were identified by Whole-exome sequencing (WES) and confirmed by Sanger sequencing.

**Interventions:**

The patient received 1 shot of IV diazepam 0.3 mg/kg after IV cannulation, followed by IV ceftriaxone 500 mg twice daily, IV hydrocortisone 20 mg every 4 hours, and albuterol, ipratropium bromide, and hypertonic saline nebulizers regularly. Additionally, she was given intravenous fluids at a rate of 60 mL/h of 5% dextrose saline. She was given a 200 mg dose of phenytoin IV for seizure control. Upon clinical suspicion of a mitochondrial disease, WES was performed, followed by Sanger sequencing for confirmation of the findings.

**Outcomes:**

The patient clinically improved, with cessation of seizures, recovery from pneumonia, and confirmed diagnosis of COXPD35.

**Lessons:**

The identification of new *TRIT1* variants and the expanding phenotypic spectrum of COXPD35 provides insights into its clinical and genotypic characteristics. WES and Sanger Sequencing confirm the diagnosis of COXPD35; however, it can be challenging in resource-limited settings.

## 1. Introduction

Combined oxidative phosphorylation deficiency 35 (COXPD35) is a mitochondrial disorder inherited in an autosomal recessive pattern.^[1,2]^ It is caused by pathogenic variants in the *TRIT1* gene, located on chromosome 1p34.2. This gene encodes the enzyme tRNA isopentenyltransferase (IPT), which plays a crucial role in modifying adenosine-37 in selecting cytosolic and mitochondrial tRNAs. This modification is essential for the proper function of these tRNAs during the synthesis of key mitochondrial proteins.^[3]^ COXPD35 is a rare disorder, with only 16 reported cases of 12 different types of allelic variants in the *TRIT1* gene in the literature up to September 2025.^[2–11]^ While no typical age of onset was identified, symptoms can start in early infancy, ranging from 3 to 14 months, although patients at a younger age have been reported.^[3]^ Although rare, the phenotype of COXPD35 has become increasingly recognized. Reported cases typically present in infancy with global developmental delay, congenital microcephaly, hypotonia, intractable seizures, and variable dysmorphic, visual, or systemic abnormalities.^[2–4]^

Given the rarity of this condition, thorough reporting of COXPD35 cases is crucial for updating the literature on this condition and providing insights into its detection and management, particularly in resource-limited settings. The first 2 cases of COXPD35 were reported in 2014 by Yarham et al.^[5]^ Here, we describe the first reported case of COXPD35 in Palestine, caused by novel compound heterozygous *TRIT1* variants that have not been previously described in the literature. This report adds to the limited global data on COXPD35 and highlights the value of whole-exome sequencing (WES) and Sanger sequencing in diagnosing rare mitochondrial diseases.

## 2. Case presentation

The 32-month-old female patient, born via an uncomplicated vaginal delivery at 39 weeks of gestation to a non-consanguineous family, was brought to the emergency department by her parents due to abnormal movements lasting more than 10 minutes. She had a 3-day history of cough, low-grade fever (37.7 °C), and increased work of breathing. Her past medical history included recurrent seizures beginning at 3 months of age, several episodes of status epilepticus, repeated admissions for bronchiolitis and aspiration, global developmental delay, failure to thrive (FTT), congenital microcephaly, and dysmorphic facial features. There is no known family history of complex neurodevelopmental disorders or genetic diseases. And she is the first and only child in this family. A metabolic disorder had been previously suspected but remained undiagnosed due to limited access to genetic testing.

## 3. Initial assessment and physical examination

On arrival, she was unresponsive with oxygen saturation of 89%, respiratory rate 29/minute, temperature 37.8 °C, blood pressure 105/68 mm Hg, and heart rate 112/minute. Status epilepticus was diagnosed and resolved after intravenous (IV) diazepam 0.3 mg/kg. Anthropometric measurements showed weight and head circumference below the 3rd percentile, with height at the 10th percentile.

**Figure 1.**
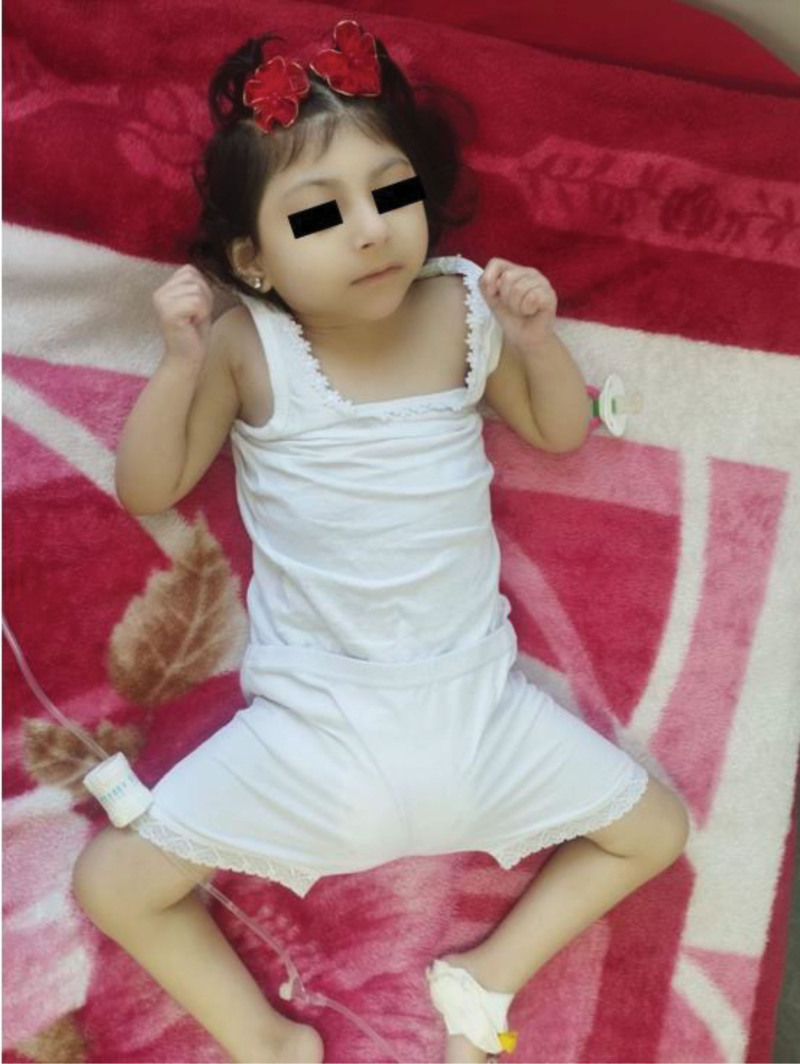
An image of the patient demonstrating the facial dysmorphism, including microcephalus, low-set ears, and microretrognathia.

Dysmorphic features included bilateral ptosis and exophthalmos, low-set ears, widely spaced teeth, and microretrognathia (Fig. 1). Chest examination revealed mild respiratory distress with bilateral wheezing. Neurological examination demonstrated increased tone, spasticity, brisk reflexes (more prominent in lower limbs), clonus, and bilateral Babinski sign. Ophthalmological evaluation confirmed bilateral ptosis and exophthalmos. Other systemic examinations were unremarkable.

The parents reported feeding difficulties (coughing/choking, prolonged feeding times) and recurrent diarrhea. The pediatric team was consulted, and based on the signs and symptoms, a chest infection was suspected, on the grounds of an undiagnosed congenital condition.

## 4. Initial investigations and diagnostic workup

**Figure 2.**
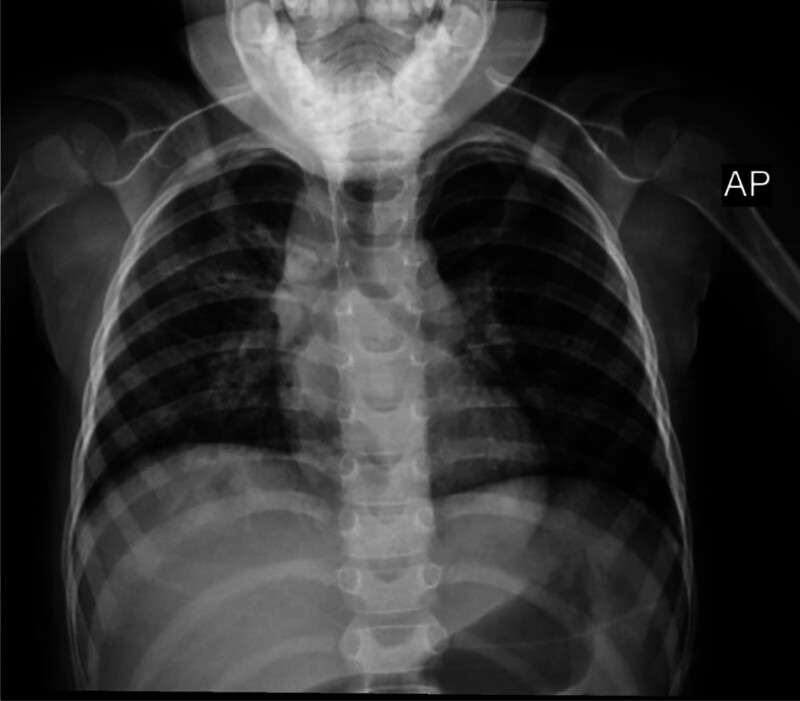
AP chest x-ray taken while the patient was in the supine position, showing right lower lobe opacities, particularly in the right retrocardiac region, suggesting airspace disease, mostly consistent with right lower lobe pneumonia. AP = anteroposterior.

Laboratory findings showed microcytic anemia with hemoglobin (Hb) 9.2 g/dL (reference range: 11.5–13.5 g/dL) and mean corpuscular volume 62.3 fL (reference range: 71–83 fL), normal inflammatory markers except a slightly elevated C-reactive protein (CRP) 8.10 mg/L (reference range: <3 mg/L), normal renal and hepatic panels, and mild electrolyte abnormalities. Chest X-ray demonstrated right lower lobe airspace opacities consistent with pneumonia (Fig. 2).

## 5. Management

The patient was admitted and treated with IV ceftriaxone 500 mg twice daily, hydrocortisone IV 20 mg every 4 hours for severe inflammatory response, albuterol, ipratropium bromide, and hypertonic saline nebulizers regularly, antiepileptic therapy (phenytoin 200 mg IV), and maintenance IV fluids with 60 mL/h of 5% dextrose saline. A second brief seizure episode on the ward resolved with IV diazepam.

## 6. Further diagnostic workup

Endocrine and metabolic evaluations—including thyroid function, parathyroid hormone, vitamin B12, folate, ferritin, haptoglobin, lipid panel, and blood gases—were largely normal, except for low serum iron (30 μg/dL, reference range: 50–120 μg/dL), consistent with microcytic anemia. Further metabolic screening tests, such as urine organic acids, plasma/urine acylcarnitine, amino acids profile, pyruvic acid, and ammonia, were not available in this hospital; therefore, they were not performed. Serum lactate dehydrogenase (LDH) was normal at 299 U/L (reference range: 110–500 U/L), and the non-fasting triglyceride level was high, 207 mg/dL (reference range: <75 mg/dL). Additionally, arterial blood gas analysis revealed a mildly elevated pH of 7.47, a decreased pCO_2_ of 31 mm Hg, bicarbonate at 23.2 mmol/L, a normal pO_2_ of 89 mm Hg, and a normal anion gap and lactate level. Suggesting mild respiratory alkalosis with compensation.

**Figure 3.**
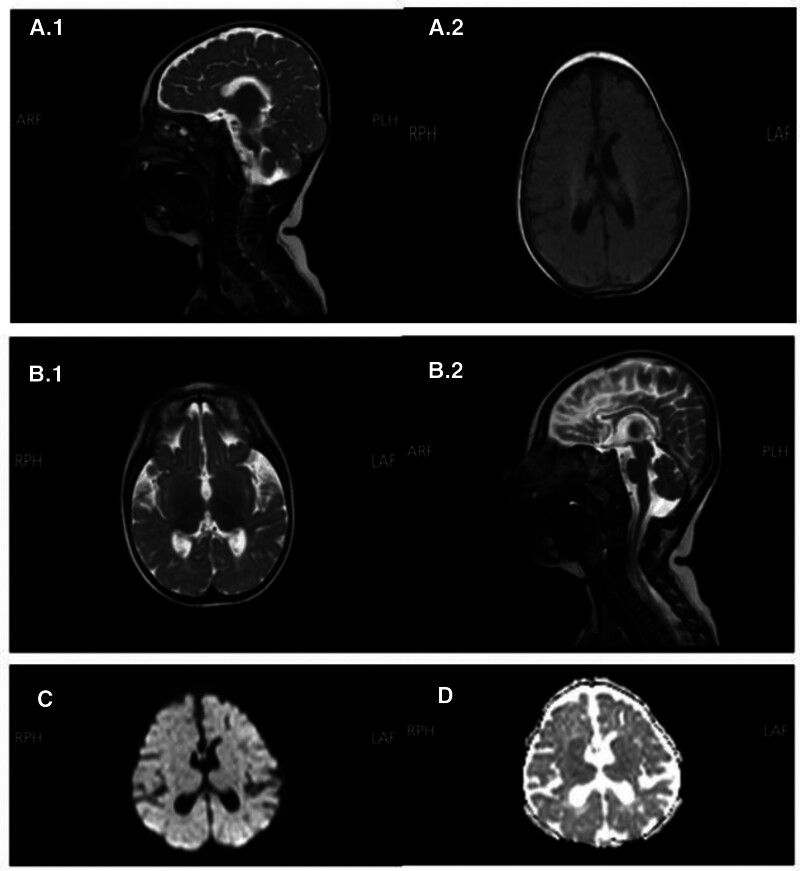
Brain MRI showing a thinning of the corpus callosum, delayed myelination for the patient’s age, and hypoplasia of the anterior horn of the right lateral ventricle. (A.1) T1W MRI sequence in sagittal section, (A.2) T1W MRI sequence in transverse section, (B.1) T2W MRI sequence in sagittal section, (B.2) T2W MRI sequence in transverse section, (C) DWI MRI sequence, and (D) ADC MRI sequence. ADC = apparent diffusion coefficient, MRI = magnetic resonance imaging.

An abdominal and pelvic ultrasound (US) was done and revealed minimal right-sided hydronephrosis, with no other abnormalities observed on the imaging. A Brain magnetic resonance imaging, previously performed, was reviewed and showed a thinning of the corpus callosum, delayed myelination for her age, and hypoplasia of the anterior horn of the right lateral ventricle (Fig. 3). A report of a previously performed electroencephalogram (EEG) was reviewed, which revealed diffuse slowing with bilateral multifocal epileptiform discharges, more prominent on the right side. The patient’s pediatric neurologist was consulted and provided the pediatric team with a management plan for her intractable seizures. Based on the clinical manifestations, examination, and laboratory results, the pediatric neurologist suspected the patient had a neurometabolic disorder. However, the diagnosis required genetic testing to be confirmed.

Following the patient’s improvement, she was discharged on day 5 following admission, on oral levetiracetam (25 mg/kg/day divided BID; 2.5 mL twice daily) and oral carbamazepine (10–20 mg/kg/day divided BID; 3 mL twice daily), alongside rectal diazepam as needed for seizure control. A follow-up visit to the outpatient pediatric clinic was arranged within 1 week. At the clinic, the patient was doing well; her pneumonia had resolved, and there was no recurrence of her seizures. Upon questioning, her mother was giving her the medications as prescribed, with no apparent adverse events. The parents were advised to perform genetic testing by WES and Sanger sequencing. However, these investigations are only available at a tertiary center in Jerusalem, which was inaccessible and beyond the parents’ financial capacity. Therefore, the final diagnosis was further delayed.

## 7. Genetic testing and final diagnosis

One month after discharge, the patient underwent WES at Al-Makassed Hospital in Jerusalem. Sequencing was performed using the GRCh38 reference genome and demonstrated high-quality coverage (mean depth 112×, with 98.4% of targeted bases covered at ≥ 20×). Variant calling was conducted using GATK v4.2, and annotation was performed with Ensembl VEP, incorporating data from gnomAD (v3.1), ClinVar (2025), and other relevant databases. Standard quality thresholds (DP >10, GQ >20, MQ >40, QD >2, FS <30) were applied, and variants were prioritized based on rarity, predicted functional impact, inheritance pattern, and relevance to mitochondrial disease. This analysis identified 2 *TRIT1* variants consistent with an autosomal recessive disorder: NM_017646.6:c.414 + 1G > A, a canonical splice-donor variant classified as pathogenic according to ACMG–AMP criteria (PVS1, PM2, PP3, PP1) due to its predicted disruption of the essential + 1 splice position, complete absence from population databases, strong in-silico support from SpliceAI (Δscore 0.99), and correct segregation from the father; and NM_017646.6:c.1205G > C, p.(Arg402Pro), a missense variant classified as a variant of uncertain significance, supported by PM2 and PP3 due to its absence from gnomAD and ClinVar and deleterious computational predictions (REVEL 0.71, CADD 27.3), with maternal inheritance consistent with recessive disease. Both variants affect evolutionarily conserved residues within functional regions of the *TRIT1* protein. Copy-number variant (CNV) analysis was performed using a read-depth–based CNV calling method integrated within the next-generation sequencing WES pipeline. No pathogenic or likely pathogenic CNVs were identified. Owing to resource constraints, functional validation studies, including ribonucleic acid (RNA) analysis or reverse transcription polymerase chain reaction (RT-PCR), were not conducted to experimentally confirm the predicted splicing effect of the NM_017646.6:c.414 + 1G > A variant. Parental segregation was confirmed by Sanger sequencing, establishing compound heterozygosity in the proband. Although the reviewers suggested attaching the Sanger chromatograms, this was unfortunately not possible because the confirmatory sequencing was performed at a different institution that did not provide the raw chromatogram files. Taken together, the clinical phenotype and molecular findings support a final diagnosis of Combined Oxidative Phosphorylation Deficiency 35 (COXPD35) due to novel compound heterozygous *TRIT1* variants. Table 1 summarizes the timeline of events in this case.

**Table 1:** Clinical timeline of events in this case of COXPD35.

Timeline	Event
August, 2022	Birth of the patient, detection of microcephaly.
November, 2022	The first seizure developed at the age of 3 mo.
November 2022–February 2025	Multiple hospitalizations due to intractable seizures and respiratory infections.
May, 2023	Suspicion of metabolic or mitochondrial disorders, lack of definitive diagnosis due to limited access to genetic testing.
March, 2025	Hospitalization due to status epilepticus.
March, 2025	Management and adjustment of antiepileptic medications.
April, 2025	Genetic testing and diagnosis of COXPD35 with WES confirmed by Sanger sequencing.

WES = whole-exome sequencing, COXPD35 = combined oxidative phosphorylation deficiency 35.

## 8. Discussion

We report the first identified case of COXPD35 in Palestine. This patient, who presented with a lobar pneumonia, status epilepticus, microcytic anemia, FTT, and an unconfirmed diagnosis of a metabolic condition based on her clinical signs and symptoms, was finally diagnosed with COXPD35 by genetic testing through WES and confirmed by Sanger sequencing. COXPD35 has not been previously reported from Palestine, based on the available literature. This may be due to the rarity of this condition, limited resources for diagnosis, or financial constraints that prevent conducting the appropriate genetic testing. Therefore, it was essential to report the first identified case of COXPD35 in Palestine.

Clinical features of this disorder include multisystemic manifestations. The central nervous system is usually affected by the disorder, with symptoms including global developmental delay, intellectual disability, intractable seizures, and truncal hypotonia.^[2,9]^ Additional features include FTT, congenital microcephaly, dysmorphic facial features, and visual disturbances.^[2,9]^ New features such as strabismus and bicuspid aortic valve were also reported in a recent case report; however, it is difficult to link them to the disorder currently.^[9]^ Most of these clinical features, reported in the literature, were present in this patient, including the global developmental delay, FTT, seizures, congenital microcephaly, dysmorphic facial features, and visual disturbances. Table 2 summarizes the clinical features in this case, in comparison with the reported cases of COXPD35 in the literature.

**Table 2:** Summary of genotypic spectrum of TRIT1 gene and clinical features of COXPD35 reported in the literature.

Clinical Feature	TRIT1 variant(s)	Age	Age of onset	Sex	Country	Consanguinity	Developmental delay	Failure to thrive	Microcephaly	Seziure	Febrile convulsion	Intellectual disability	Hypotonia	Spasticity	Other Facial Features	Hearing	Vision	Gastrointestinal evaluation	Cardiac evaluation	Endocrinology	Respiratory	Brain MRI	EEG
This Case (2025)	Compound heterozygous: C.414 + 1G > A (likely pathogenic), C.1205G > C p.Arg402Pro	2.5 yr	3 mo	Female	Palestine	No	Global	Yes	Yes	Yes	Yes	Yes	No	N/A	Low-set ears and microretrognathia	N/A	Bilateral ptosis and exophthalmos, and history of bilateral closed-angle glaucoma.	Feeding difficulties, recurrent aspiration and diarrhea	N/A	Normal	Recurrent aspiration and respiratory infections	Abnormal, thinning of the corpus callosum, delayed myelination for the patient’s age, and hypoplasia of the anterior horn of the right lateral ventricle.	Abnormal
Yarham JW et al (Case 1) (2014)^[5]^	Homozygous c.968G.A (p.Arg323Gln)	N/A	N/A	Female	UK-Pakistani	Yes	Yes	N/A	Yes	Yes	Yes	No	No	N/A	N/A	Normal	Normal	N/A	Normal	Diabetes	N/A	Normal	Abnormal
Yarham JW et al. (Case 2) (2014)^[5]^	Homozygous c.968G.A (p.Arg323Gln)	16 yr	13.5 mo	Male	UK-Pakistani	Yes	Yes	N/A	Yes	Yes	Yes	Yes	No	N/A	N/A	Normal	Normal	N/A	Normal	Diabetes	N/A	Normal	Abnormal
Whittaker et al (Case 3) (2015)^[7]^	Homozygous c.968G.A (p.Arg323Gln)	N/A	N/A	N/A	N/A	N/A	N/A	N/A	N/A	Yes	N/A	N/A	N/A	N/A	N/A	N/A	N/A	N/A	N/A	N/A	N/A	N/A	Abnormal
Yoo et al (Case 4) (2021)^[2]^	Compound heterozygous: c.979G > A (p.Glu327Lys) and c.682 + 2 T > C	13 yr	3 mo	Male	Korea	No	Yes	Yes	Yes	Yes	N/A	Yes	N/A	N/A	N/A	N/A	Optic disc hypoplasia	N/A	N/A	Transient subclinical hypothyroidism, hypertriglyceridemia, and vitamin D deficiency	N/A	Hydrocephalus and Dandy-Walker syndrome	Abnormal
Yoo et al (Case 5) (2021)^[2]^	Compound heterozygous: c.979G > A (p.Glu327Lys) and c.682 + 2 T > C	16 yr	8 mo	Female	Korea	No	Yes	Yes	N/A	Yes	N/A	Yes	N/A	Yes	N/A	N/A	No visual acuity due to cataract or optic disc hypoplasia.	N/A	N/A	N/A	N/A	Reduced volume of the periventricular white matter around the temporal horn, megacisterna magna, and diffuse thinning of the corpus callosum	Abnormal
Kernohan et al (Case 6) (2017)^[6]^	Compound heterozygous: c.1256A > C (p.His419Pro), and c.848T > G (p.Ile283Ser)	4 yr	3 mo	Female	USA	No	Yes	Yes	Yes	Yes	Yes	Yes	Yes	N/A	N/A	Normal	Optic disc hypoplasia	Normal	ASD	Elevated TSH with normal T4	N/A	Septo-optic dysplasia, partial agenesis of corpus callosum	Abnormal
Kernohan et al. (Case 7) (2017)^[6]^	Compound heterozygous: c.1256A > C (p.His419Pro)/ c.1204C > T (p.Arg402Ter)	9 yr	6 mo	Female	Canada	No	Yes	Yes	Yes	Yes	Yes	Profound	Yes	N/A	N/A	Normal	Normal structure/No tracking	G-tube: age 4	ASD and VSD	Normal	N/A	Generalized cerebral atrophy	Abnormal
Kernohan et al (Case 8) (2017)^[6]^	Compound heterozygous: c.856A > G (p.Lys286Glu), and c.22C > T (p.Arg8Ter)	N/A	5 mo	Female	USA	No	Yes	Yes	Yes	Yes	Yes	N/A	Yes	N/A	N/A	Normal	Myopia, astigmatism, left esotropia	Constipation	N/A	N/A	N/A	Frontal atrophy and increased fluid	Abnormal
Kernohan et al (Case 9) (2017)^[6]^	Compound heterozygous: c.856A > G (p.Lys286Glu), and c.22C > T (p.Arg8Ter)	N/A	N/A	Male	USA	No	N/A	N/A	Yes	Yes	N/A	N/A	N/A	N/A	N/A	N/A	N/A	N/A	N/A	N/A	N/A	N/A	N/A
Balciuniene et al (Case 10) (2019)^[8]^	Compound heterozygous: c.334del (p.Arg112fs), and c.326T > C (p.Ile109Thr)	N/A	N/A	N/A	USA	N/A	N/A	N/A	N/A	Yes	N/A	N/A	N/A	N/A	N/A	N/A	N/A	N/A	N/A	N/A	N/A	N/A	N/A
Takenouchi et al (Case 11) (2019)^[10]^	Compound heterozygous: c.244A > G p.(Met82Val) and c.1034A > G p.(Tyr345Cys)	N/A	14 mo	Female	Japan	No	Yes	Yes	No	Yes	N/A	N/A	Yes	N/A	N/A	N/A	N/A	N/A	N/A	N/A	N/A	Normal	Abnormal
Forde et al (Case 12) (2020)^[3]^	Compound heterozygous: c.1171_1172del; (p.Lys391Glufs*7), and c.334del; (p. Arg112Glufs*36)	N/A	Antenatal	Female	Romania	Yes	Yes	Yes	Yes	Yes	N/A	Yes	Yes	Yes	N/A	Bilateral SNHL	Optic nerve hypoplasia and pigmentary retinopathy	Feeding difficulties, poor growth	ASD	N/A	Recurrent respiratory infections	Diffuse cortical atrophy, hypomyelination, thin corpus callosum and abnormal cerebellar vermis, in addition to polymicrogyria	Abnormal
Yildirim et al (Case 13) (2022)^[9]^	Homozygous missense variant of c.246G > C (p.Met82Ile)	6 yr	6 mo	Male	Turkey	Yes	Yes	Yes	Yes	Yes	Yes	Yes	Yes, truncal hypotonia	Yes	Telorism, epicanthal fold, prominent ear, high-arched palate, mild retrognathia	Normal	Strabismus	Malnutrition, constipation	Bicuspid aortic valve	Polyuria and polydipsia, ketotic hypoglycemia.	N/A	Normal	Abnormal
Muylle et al (Case 14) (2022)^[4]^	Homozygous:c.967C > T;[882_883del], (p.Arg323Tyr.)	1 yr	3 mo	Female	China	No	Yes	N/A	Yes	Yes	N/A	N/A	No	Yes	N/A	Normal	Esotropia	Normal	Normal	Normal	N/A	Delayed myelination, corpus callosum dysplasia, and an enlarged cisterna magna.	Abnormal
Muylle et al (Case 15) (2022)^[4]^	Compound heterozygous: c.326T > C (p.Ile109Thr) and c.979 C > T (p.Arg327*).	3 yr	4 mo	Male	China	No	Yes	N/A	Yes	Yes	N/A	N/A	Yes	Yes	N/A	Recurrent earinfections	Strabismus	N/A	N/A	N/A	N/A	Brain atrophy	Abnormal
Aaltio et al (Case 16) (2024)^[11]^	Compound heterozygous: c.70C > T (p.Pro24Ser), and c.979C > T (p.Arg327Ter).	7 yr	9 mo	Female	Finnland	No	Yes	N/A	Yes	Yes	N/A	N/A	N/A	Yes	N/A	N/A	Nystagmus	N/A	N/A	N/A	N/A	Thin corpus callosum and white matter abnormality.	N/A

COXPD35 = combined oxidative phosphorylation deficiency 35, TRIT1 = enzyme tRNA isopentenyltransferase 1 gene.

By September 2025, only 16 cases of COXPD35 have been reported in the literature; however, we expect the actual number would be higher, with many undetected cases. Challenges to the diagnosis, especially in low- to middle-income countries, included the rarity of this condition, limited resources, and financial constraints that hinder the conduct of the appropriate genetic testing. On the other hand, this condition can be diagnosed through noninvasive tests using peripheral blood and urine samples.^[6,8,10]^ WES serves as an efficient diagnostic tool to identify de novo gene variants and aid in the diagnosis of children with heterogeneous neurological disorders.^[11]^ We recommend that clinicians suspect COXPD35 and other mitochondrial diseases based on clinical features, and use WES to confirm the diagnosis and enhance the detection of COXPD35 cases.

As the phenotypic variations of COXPD35 continue to expand, its genotype is expanding similarly, with novel variants of the *TRIT1* gene continuously identified and reported in the literature. The clinical features, which are similar to those reported in previous cases, further suggest that these variants are linked to COXPD35. Here, we report 2 novel heterozygous *TRIT1* variants—c.414 + 1G > A and c.1205G > C, p.(Arg402Pro)–associated with COXPD35. As of September 2025, neither variant had been reported previously in the literature, ClinVar, or gnomAD, supporting their novelty. The splice-site variant c.414 + 1G > A affects the canonical donor site. It is predicted to result in aberrant mRNA splicing and complete loss-of-functional protein, consistent with a pathogenic loss-of-function allele. In contrast, c.1205G > C, p.(Arg402Pro) is a missense variant that introduces a nonconservative substitution in the enzyme’s catalytic domain. This may alter local conformation and substrate interaction, though functional studies are needed to confirm its effect. Together, these variants likely result in compound heterozygosity, leading to defective tRNA isopentenylation, impaired mitochondrial translation, and secondary oxidative phosphorylation dysfunction, typical of COXPD35.

Managing COXPD35 can be challenging due to the rarity of the condition and the lack of specific therapeutic options. Clinicians usually focus on managing seizures with antiepileptic medications and other measures of conservative treatment. Previous studies have reported a potential benefit of tauroursodeoxycholic acid (TUDCA) in regulating mitochondrial and cytosolic protein homeostasis, through taurine modification of mitochondrial tRNA (mt-tRNA).^[10,12]^ This was shown to suppress the cytotoxicity in cells with dismodified mt-tRNA, and serves as a potential therapeutic target for such mitochondrial disorders.^[10,12]^ TUDCA is currently FDA-approved for treating cholestatic liver diseases, and further clinical evidence is needed to confirm its efficacy in treating COXPD35 and other mitochondrial disorders.^[10,12]^

## 9. Conclusion

We report the first case of COXPD35 in Palestine. This 2-year-and-6-month-old female patient with seizures, neurodevelopmental delay, microcephaly, and dysmorphic facial features was diagnosed clinically and confirmed by WES. While clinical suspicion is essential for diagnosing COXPD35, WES and Sanger Sequencing help clinicians detect cases of rare mitochondrial diseases; however, it can be challenging in resource-limited settings.

## Author contributions

**Conceptualization:** Ali A.A. Doudin, Weam N.I. Omran, Saja M. Alkomi, Ahmad Rjoub, Hamza Shoubaki, Shurooq Tayseer Abu Mufreh.

**Data curation:** Ali A.A. Doudin, Weam N.I. Omran, Saja M. Alkomi, Ahmad Rjoub, Hamza Shoubaki, Shurooq Tayseer Abu Mufreh.

**Supervision:** Ahmad Rjoub, Hamza Shoubaki.

**Writing – original draft:** Ali A.A. Doudin, Weam N.I. Omran, Saja M. Alkomi, Ahmad Rjoub, Shurooq Tayseer Abu Mufreh.

**Writing – review & editing:** Ahmad Rjoub, Hamza Shoubaki.
